# Preoperative Diagnosis of Gallbladder Volvulus: A Case Report

**DOI:** 10.7759/cureus.114276

**Published:** 2026-08-10

**Authors:** George Asfour, Raneem Bader, Barak Bar-Zakai, Harbi Khalayleh

**Affiliations:** 1 Division of Surgery, Kaplan Medical Center, The Hebrew University Faculty of Medicine, Rehovot, ISR; 2 Division of Surgery, Assuta Medical Center, Ashdod, ISR

**Keywords:** abdominal ct, acute volvulus, gallbladder torsion, gallbladder volvulus, lap cholecystectomy

## Abstract

Gallbladder volvulus is a rare condition caused by rotation of the gallbladder around the cystic duct and cystic artery. Because its clinical and radiological features often overlap with those of acute cholecystitis, preoperative diagnosis remains challenging, with historically only approximately 10% of cases recognized before surgery. We report the case of an 84-year-old woman who presented with a four-day history of epigastric pain radiating to the right upper quadrant. Abdominal computed tomography (CT) demonstrated a markedly distended gallbladder displaced completely outside its hepatic fossa, consistent with a floating gallbladder. Additional findings included mural thickening with absent enhancement, suggesting vascular compromise, and a twisted cystic pedicle producing a characteristic whirl sign. Together, these findings raised strong preoperative suspicion for gallbladder volvulus rather than routine acute cholecystitis. Emergency laparoscopy confirmed complete torsion of a gangrenous gallbladder. Detorsion followed by laparoscopic cholecystectomy was performed, and the patient recovered without postoperative complications. This case highlights the diagnostic importance of recognizing the combination of a floating gallbladder, absent mural enhancement, and a twisted cystic pedicle. Early identification of these CT findings may facilitate prompt surgical intervention and reduce the risk of ischemic complications.

## Introduction

Gallbladder volvulus (GV), also known as gallbladder torsion, is a rare but important differential diagnosis in patients presenting with acute right upper quadrant pain [1-9]. It is caused by axial rotation of the gallbladder around the cystic duct and cystic artery, which may compromise bile drainage and vascular supply [4,6,9]. If not recognized promptly, it can progress to gallbladder ischemia, gangrene, perforation, and biliary peritonitis [2,4,9].

GV was first described by Wendel in 1898 [2,4,8]. More recently, nearly 500 cases have been reported in the literature [2,8,9]. Although it can occur at any age, it is most commonly reported in elderly patients, particularly women in the seventh and eighth decades of life [2,5,6,8,9]. Reported predisposing factors include advanced age, female sex, liver atrophy, loss of visceral fat, and an elongated gallbladder mesentery, which may result in a mobile or “floating” gallbladder [2,4,9].

Preoperative diagnosis remains challenging because GV often presents with clinical features similar to acute cholecystitis, including acute right upper quadrant pain, nausea, vomiting, and nonspecific laboratory findings [4,8,9]. Historically, only about 10% of reported GV cases have been diagnosed preoperatively, although more recent reviews suggest that this rate has improved with the increased use of advanced imaging [5,9]. Therefore, distinguishing GV from routine acute cholecystitis before surgery is clinically important [2,4,5,9].

Although radiological findings may be nonspecific, certain imaging features should raise suspicion for GV. These include displacement of the gallbladder from its hepatic fossa, a “floating” gallbladder, wall non-enhancement suggesting vascular compromise, and the “whirl sign” of a twisted cystic pedicle [2,3,4,9]. Recognition of these findings can facilitate early surgical planning and prompt laparoscopic management [1,2,5].

Herein, we report a case of GV in an elderly female patient in whom characteristic computed tomography (CT) findings enabled preoperative diagnosis and prompt laparoscopic treatment. The case is presented in accordance with the CARE and SCARE reporting frameworks [10,11].

## Case presentation

Patient information and clinical findings

An 84-year-old woman arrived at the emergency department at 11:00 am on September 3, 2021, after four days of epigastric pain radiating to the right upper quadrant. The pain had intensified on the day of presentation and was accompanied by marked nausea and vomiting. She was alert, cognitively intact, and independent in daily activities. No formal frailty score had been recorded. Her height was 163 cm, weight 58 kg, and body mass index 21.8 kg/m². She was assigned American Society of Anesthesiologists (ASA) physical status II.

Her medical history included hypertension, dyslipidemia, lumbar spondylosis, and chronic hepatitis C virus infection. The available record described chronic hepatitis C without cirrhosis; hepatitis serology or viral-load testing was not repeated during this admission. Regular medications were amlodipine 5 mg once daily, aspirin 75 mg once daily, levothyroxine 100 µg, and omeprazole 20 mg once daily. Previous abdominal surgery consisted of hysterectomy with salpingectomy in 2002.

At presentation, she was afebrile and hemodynamically stable. Examination demonstrated epigastric and right upper quadrant tenderness with guarding. Her vital signs in the emergency department and preoperatively are presented in Table 1.

**Table 1:** Vital signs at emergency department presentation and before surgery. SBP = systolic blood pressure; SpO₂ = peripheral oxygen saturation

Time point	Temperature, °C (reference range: 36.0–37.5)	Blood pressure, mmHg (clinical target SBP ≥90 mmHg)	Heart rate, beats/minute (reference range: 60–100)	SpO₂, % (reference range: ≥95)
Emergency department arrival	36.3	115/51	67	96
Preoperative	36.4	118/61	68	95

Admission laboratory testing demonstrated mild neutrophilic leukocytosis (white blood cell count 12.2 × 10⁹/L; neutrophils 87%), mild hyponatremia (134 mEq/L), and an elevated C-reactive protein level (2.60 mg/dL), with preserved renal function and normal total bilirubin, alanine aminotransferase, and alkaline phosphatase levels; laboratory values at admission and postoperatively are summarized in Table 2.

**Table 2:** Laboratory findings during hospitalization. Laboratory values are presented at admission, during the early postoperative period, and on postoperative day one. Reference intervals are those provided by the institutional laboratory. ALP = alkaline phosphatase; ALT = alanine aminotransferase; AST = aspartate aminotransferase; CRP = C-reactive protein; GGT = gamma-glutamyl transferase; WBC = white blood cell count

Laboratory variable	Reference interval	Admission (September 3)	Early postoperative (September 4)	Postoperative day 1 (September 5)
WBC (×10⁹/L)	4.50–11.00	12.20	11.80	7.40
Neutrophils (%)	40.0–70.0	87.0	88.0	70.2
Hemoglobin (g/dL)	12.0–16.0	12.1	11.8	11.4
Platelets (×10⁹/L)	150–450	155	135	152
Creatinine (mg/dL)	0.57–1.11	0.63	0.59	0.66
Sodium (mEq/L)	136–145	134	136	136
Potassium (mEq/L)	3.4–5.2	3.9	3.7	4.2
Total bilirubin (mg/dL)	0.3–1.2	1.1	2.4	0.6
Direct bilirubin (mg/dL)	≤0.5	NR*	1.4	0.3
AST (U/L)	5–34	NR*	117	41
ALT (U/L)	0–55	24	93	58
ALP (U/L)	40–150	99	133	108
GGT (U/L)	9–36	51	96	72
CRP (mg/dL)	0.00–0.50	2.60	6.56	6.40

Diagnostic assessment

**Figure 1:**
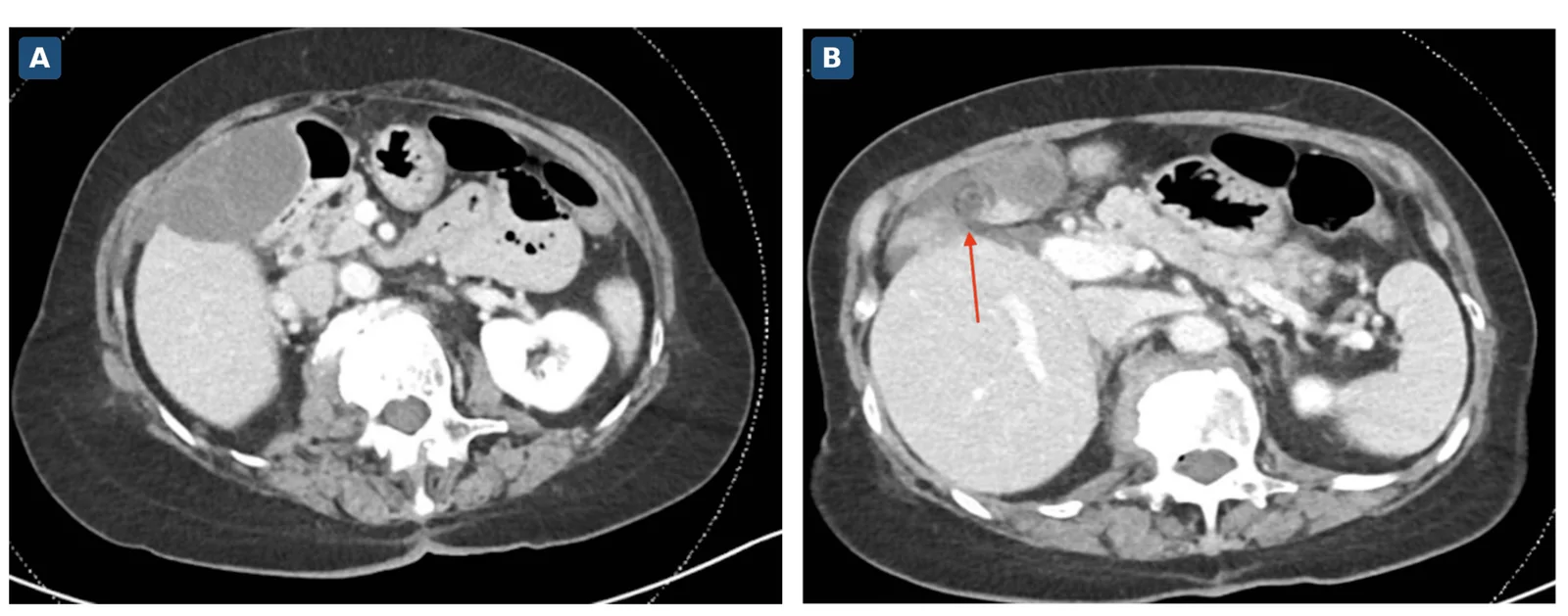
Intravenous contrast-enhanced abdominal computed tomography. (A) Distended gallbladder displaced from its normal fossa and situated anterior and lateral to the liver. (B) Twisting of the cystic pedicle, the whirl sign, is indicated by the red arrow, with reduced mural enhancement and wall irregularity.

Abdominal CT demonstrated a markedly distended “floating” gallbladder displaced outside its normal anatomical fossa (Figure 1A). In contrast to typical acute cholecystitis, in which the gallbladder usually remains within the hepatic fossa and demonstrates inflammatory wall enhancement, our patient had a distended gallbladder, measuring 63.1 × 46.2 mm, with extra-fossa displacement, wall non-enhancement suggesting vascular compromise, wall thickening, and a “whirl sign” of the twisted cystic pedicle (red arrow, Figure 1B). The common bile duct was described as non-dilated. Together, these findings raised strong preoperative suspicion for GV, which was confirmed at laparoscopy.

Treatment and operative findings

Initial treatment consisted of intravenous fluids, analgesia, and broad-spectrum antibiotics, followed by urgent operative management. Laparoscopic cholecystectomy began at 03:30 am on September 4, 2021, 16.5 hours after emergency department arrival. The patient was classified as ASA II, remained hemodynamically stable throughout anesthesia and surgery, and did not require vasopressor support.

**Figure 2:**
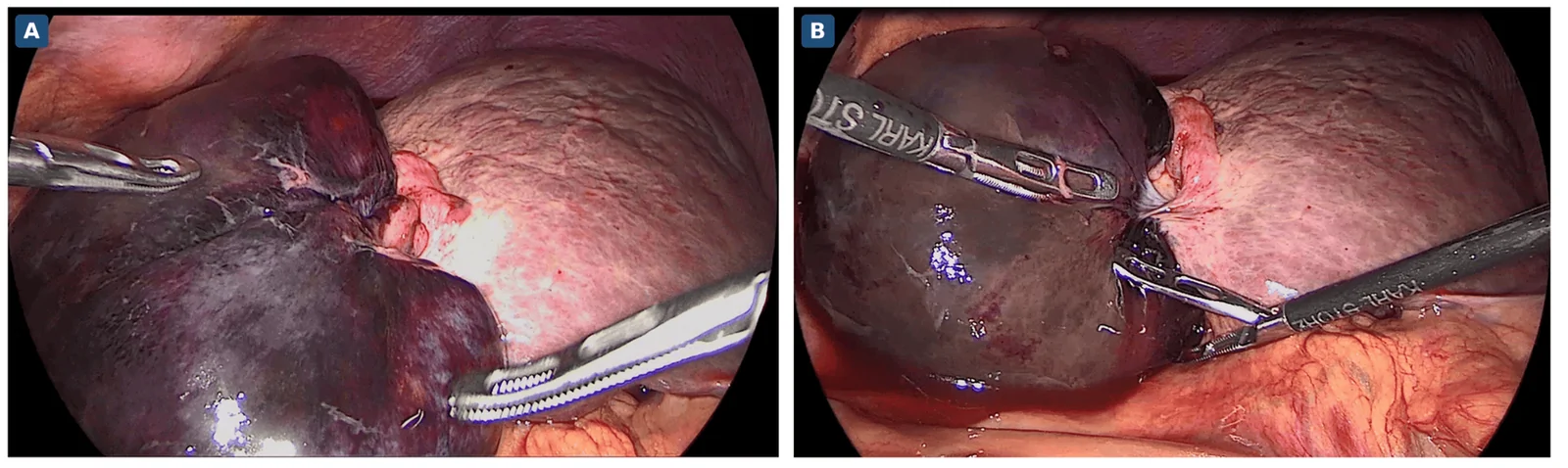
Laparoscopic findings. (A) Intraoperative visualization of gallbladder volvulus. Image showing a distended, gangrenous gallbladder with a full twist. (B) Intraoperative visualization of gallbladder volvulus. During the untwisting process, the gallbladder reveals a complete long mesentery between the gallbladder and the liver.

Laparoscopy demonstrated a markedly distended, ischemic-appearing gallbladder outside its usual fossa and a complete volvulus of more than 180° around the cystic duct and cystic artery (Figure 2A). The gallbladder was first detorsed, exposing a long mesentery between the gallbladder and liver (Figure 2B), and laparoscopic cholecystectomy was then completed. The anatomy was descriptively consistent with a highly mobile or floating gallbladder, but a formal Gross attachment type was not assigned. Blood loss was documented as negligible, without a numeric estimate, and no intraoperative complication occurred.

A suction drain was placed in the right subhepatic space according to the department’s routine practice after a complicated cholecystectomy. Antibiotics were administered perioperatively and were not continued postoperatively.

Outcome and follow-up

The inpatient postoperative course was uncomplicated. Oral intake was resumed and tolerated on postoperative day one, and the drain was removed before discharge. The patient was discharged home in good general condition on September 5, 2021, postoperative day one. No perioperative adverse event was documented. Final histopathology demonstrated transmural gallbladder necrosis. No outpatient follow-up occurred after discharge.

Clinical timeline

The key events from the patient’s presentation through diagnosis, surgical management, and hospital discharge are summarized chronologically in Table 3.

**Table 3:** Clinical timeline from symptom onset through discharge.

Date/time	Clinical event
August 30 to September 3	Approximately four days of epigastric pain radiating to the right upper quadrant, with nausea and vomiting
September 3, 11:00 am	Emergency department arrival; afebrile and hemodynamically stable; right upper quadrant tenderness and guarding; laboratory and CT assessment
September 3-4	CT prospectively raised suspicion of gallbladder volvulus; intravenous fluids, analgesia, and antibiotics were administered; urgent surgery arranged
September 4, 03:30 am	Laparoscopy confirmed complete volvulus >180°; detorsion followed by laparoscopic cholecystectomy and subhepatic drain placement
September 4-5	Stable postoperative course without documented complication; oral intake resumed; drain removed
September 5	Discharged home in good general condition on postoperative day one
After discharge	No outpatient follow-up or patient perspective available

Patient perspective

A patient-authored perspective was not available because the patient did not attend follow-up after discharge.

## Discussion

Epidemiology and pathophysiology

GV is an exceptionally rare clinical phenomenon, with a reported incidence of 1 in 365,520 hospital admissions [2,4]. Although it can occur across all age groups, it is predominantly observed in elderly women, with a reported female-to-male ratio of approximately 3:1 and the highest incidence in the seventh and eighth decades of life [2,4,6,8]. GV was first described by Wendel in 1898 as a “floating gallbladder,” and more than 500 cases have been reported in the world literature [2,8,9].

GV occurs when the gallbladder rotates along the longitudinal axis of the cystic duct and cystic artery [4,6,8,9]. This torsion can obstruct biliary drainage and compromise vascular inflow, leading to gallbladder ischemia [4,6,9]. Gallbladder torsion is classified as partial or incomplete when rotation around the cystic pedicle is less than or equal to 180° and complete when rotation exceeds 180°. Torsion may occur in either a clockwise or counterclockwise direction [2,5]. If not recognized and treated promptly, vascular compromise may progress to infarction, gangrene, perforation, bilious peritonitis, and hemodynamic instability [2,4,7,9].

Risk factors and predisposing anatomy

Most cases are considered idiopathic; however, several anatomical and clinical factors have been associated with GV. Reported predisposing factors include advanced age (>70 years), female sex, liver atrophy, weight loss, kyphoscoliosis, atherosclerosis, an elongated gallbladder mesentery, and loss of visceral fat [2,4]. Loss of visceral fat and mesenteric elongation may increase gallbladder mobility, creating a “floating” gallbladder that is more prone to torsion [2,4]. Additional contributing factors, such as congenital mesenteric anomalies, a tortuous cystic duct, atherosclerosis of the cystic artery, and forceful peristaltic movements, have also been proposed as potential contributors to torsion [2,4].

The diagnostic challenge

Preoperative diagnosis of GV remains challenging because its clinical presentation frequently overlaps with acute cholecystitis. Patients commonly present with acute right upper quadrant pain, nausea, vomiting, abdominal tenderness or guarding, and nonspecific laboratory abnormalities [2,4,9]. Historically, only approximately 10% of reported cases were diagnosed preoperatively, although this rate has improved with the increasing use of advanced imaging [5,9]. A study published by Nakao et al. reviewed 245 cases reported in the Japanese literature and identified acute abdominal pain, infrequent fever or jaundice, poor response to antibiotics, and a markedly enlarged floating gallbladder as features that may help distinguish torsion from acute cholecystitis [12]. Reilly et al., in a systematic review of 324 published cases, reported a median age of 77 years, a female-to-male ratio of 4:1, and preoperative recognition in approximately one quarter of cases reported from 1991 onward [5]. More recently, Nho et al. reported six consecutive cases and compared five contemporary patients with torsion against 1,974 patients with other acute gallbladder diseases; only two of the six torsions were suspected preoperatively, with a CT whirl sign and absent vascular flow on ultrasonography serving as the decisive imaging clues [13]. These findings closely parallel our patient, whose age, sex, normal bilirubin, and CT demonstration of a floating gallbladder, non-enhancing wall, and twisted cystic pedicle enabled diagnosis before surgery.

The diagnosis should be suspected when the clinical course or imaging findings are atypical for ordinary acute cholecystitis. A palpable right upper quadrant mass may occasionally be present, representing the distended and mobile “floating” gallbladder, although this finding can be missed because of abdominal guarding [2,4]. Laboratory studies are often nonspecific; leukocytosis and elevated C-reactive protein may be present, while liver function tests and bilirubin often remain within normal limits [2-4]. This pattern reflects the fact that torsion primarily compromises the cystic pedicle rather than causing primary obstruction of the common bile duct.

Radiographic findings

Imaging findings may be nonspecific, but they are essential for raising suspicion and narrowing the differential diagnosis [2-4,9]. Ultrasound may show a distended, “wandering,” or pedunculated gallbladder located outside its normal hepatic fossa, often with wall thickening, pericholecystic fluid, and absence of gallstones [2-4]. Additional sonographic or cross-sectional imaging clues include a cystic duct knot, swirling of the cystic vessels, thumbprinting suggestive of gangrenous change, and a floating gallbladder sign [9].

CT may demonstrate an enlarged or abnormally positioned gallbladder, displacement from the hepatic fossa, wall non-enhancement suggesting vascular compromise, and the characteristic “whirl sign” of a twisted cystic pedicle [2,3,9]. These findings are particularly important because they help distinguish GV from routine acute cholecystitis, in which the gallbladder usually remains within its hepatic fossa and demonstrates inflammatory wall enhancement. MRI may provide additional evidence of hemorrhage, infarction, or necrosis, while magnetic resonance cholangiopancreatography may demonstrate distortion or tapering of the extrahepatic bile ducts [2-4]. Hepatobiliary iminodiacetic acid scanning has also been reported to show a “bull’s-eye” appearance in GV [2,4]. Although gallstones are common in routine gallbladder disease, they are not required for GV and were reported in only approximately 24.4% of patients in one large review [2].

Clinical triad and management

A “triad of triads” has been described to support the clinical recognition of gallbladder torsion [2,4]. The characteristic patient is typically a thin, older individual, often with chronic pulmonary disease or spinal deformity, who develops sudden-onset right upper quadrant pain accompanied by early vomiting. Examination may reveal a palpable right upper quadrant mass, an absence of jaundice or marked toxemia, and a discrepancy between the pulse rate and temperature. Although these features are not diagnostic, their coexistence should increase suspicion for GV, particularly when the presentation is atypical for routine acute cholecystitis [2,4].

A high index of suspicion should be maintained when clinical, laboratory, or imaging findings are atypical for ordinary acute cholecystitis or when symptoms fail to improve despite appropriate resuscitation and antibiotic therapy [2,4]. Unlike uncomplicated acute cholecystitis, GV is a surgical emergency, and a “cooling-off” period is not appropriate once torsion is suspected [2]. Definitive treatment is urgent cholecystectomy, performed either laparoscopically or through an open approach [1,2,4,6]. Laparoscopic management is feasible and typically involves decompression when needed, detorsion, identification of the critical view of safety, and cholecystectomy [1,2]. Prompt diagnosis and surgical treatment are essential to prevent serious complications, including gangrene, perforation, bilious peritonitis, and hemodynamic instability [2,4,9].

Strengths and limitations

A principal strength of this report is the preoperative recognition of GV based on characteristic CT findings, followed by operative and histopathological confirmation. Nevertheless, as a single case report, its findings cannot be generalized. Additional limitations include the absence of outpatient follow-up and a patient-reported perspective, as well as incomplete documentation of certain radiological and operative details. Despite these limitations, the case provides a clinically relevant illustration of imaging findings that may facilitate earlier diagnosis and prompt surgical treatment.

## Conclusions

GV is an uncommon but important differential diagnosis in older, thin patients presenting with acute right upper quadrant pain, particularly when the clinical or imaging findings are atypical for routine acute cholecystitis. This case highlights the diagnostic value of characteristic CT findings, including a floating gallbladder, displacement from the hepatic fossa, absent mural enhancement, and the whirl sign of a twisted cystic pedicle. The coexistence of these findings should prompt urgent surgical consultation, even when laboratory abnormalities are modest or nonspecific. Early recognition may shorten the interval to definitive treatment and prevent progression to gallbladder ischemia, gangrene, perforation, and biliary peritonitis. Laparoscopy provides both diagnostic confirmation and definitive treatment through detorsion and cholecystectomy. Greater awareness of this characteristic imaging pattern among radiologists, emergency physicians, and surgeons may improve the rate of preoperative diagnosis. Therefore, gallbladder volvulus should remain an important diagnostic consideration whenever a markedly distended and abnormally positioned gallbladder is identified.
